# The effect of abusive supervision variability on work–family conflict: The role of psychological detachment and optimism

**DOI:** 10.3389/fpsyg.2022.973634

**Published:** 2023-01-17

**Authors:** Shuwei Wang, Xiaoxin Lin, Jun Wu

**Affiliations:** ^1^School of Management, Guangzhou Panyu Polytechnic, Guangzhou, China; ^2^Mahasarakham Business School, Mahasarakham University, Mahasarakham, Thailand; ^3^School of Management, Jinan University, Guangzhou, China; ^4^School of Innovation and Entrepreneurship, Guangzhou Panyu Polytechnic, Guangzhou, China

**Keywords:** abusive supervision variability, psychological detachment, optimism, work–family conflict, conservation of resources theory

## Abstract

Although a number of studies have examined the effects of abusive supervision variability, which refers to leaders engaging in differential abuse toward different subordinates within the team on work-related outcomes, scant research has investigated whether and how abusive supervision variability affects non-work outcomes. Drawing on the conservation of resources theory, the current study explores how abusive supervision variability affects work–family conflict through psychological detachment, as well as the moderating role of optimism. Results based on a survey of 260 employees from nine companies show that abusive supervision variability is significantly and positively related to work–family conflict. Psychological detachment mediates the effect of abusive supervision variability on work–family conflict. Optimism moderates the relationship between abusive supervision variability and psychological detachment and the indirect effects of abusive supervision variability on work–family conflict through psychological detachment. This study extends the literature on the effects of abusive supervision variability and provides several important practical implications.

## Introduction

Abusive supervision refers to leaders’ “sustained display of hostile verbal and nonverbal behaviours” toward their subordinates (Tepper, 2000). Abusive supervision costs U.S. companies an estimated $23.8 billion annually, directly affecting 13.6% of employees (Huang et al., 2022). Research has demonstrated that abusive supervision negatively affects target members’ emotions, cognition, and behavior, such as increasing targeted employees’ work pressure, causing counterproductive behaviors, reducing employees’ work performance and creativity, and improving their turnover intention (Harvey et al., 2007; Tepper et al., 2008; Mawritz et al., 2012; Jiang and Gu, 2016; Zheng and Liu, 2017; Chen and Liu, 2019; Choi et al., 2019; Jiang et al., 2019; Hu et al., 2022). This stream of research has assumed that abusive supervision occurs in a one-to-one situation. However, leaders might engage in differential abuse toward different subordinates (Hannah et al., 2013; Ogunfowora, 2013; Ogunfowora et al., 2019). For instance, leaders are less likely to abuse subordinates who have high social exchange quality but may be more abusive toward subordinates who perform poorly (Meyer et al., 1993; Harris et al., 2011; Tepper et al., 2017). This phenomenon has been referred to as abusive supervision variability (Ogunfowora, 2013).

The extant literature has largely investigated the work-related harmful effects of abusive supervision variability, such as reduced organizational citizen behavior, diminished creativity, high turnover intention (Ogunfowora, 2013; Burton, et al.,2014; Peng A. C. et al., 2014; Schaubroeck et al., 2016; Rousseau and Aubé, 2018; Ogunfowora et al., 2019; Peng et al., 2019; Shen et al., 2020), and deviant behavior (Jiang et al., 2019). However, scant attention has been given to the possible impact of abusive supervision variability on non-work outcomes, such as work–family conflict. Such an omission is surprising given that the boundary between the work and family domains is blurred, and workplace injustice (e.g., abusive supervision variability in the current study) has been regarded as an important predictor of employees’ state of mind regarding their family life.

Toward this end, this study explores whether and how abusive supervision variability affects employees’ work–family conflict. The conservation of resources theory points out that individuals tend to maintain, protect, and obtain resources. The loss of resources (whether actual or potential resources) causes them to experience tension and pressure (Hobfoll et al., 2018). Daily work events, such as leadership behavior, affect employees’ off-work psychological experience (Foulk et al., 2018; Song et al., 2018; Liao et al., 2021). Following this logic, employees who experience abusive supervision variability are likely to consume more resources to surmise the intention of leaders, making them unable to stop thinking about work-related topics during non-working hours. That is, they cannot recover the loss of psychological resources through psychological detachment. This, in turn, makes it difficult for them to devote themselves to family life, which might cause work–family conflict. Furthermore, when employees are highly optimistic, they are able to react to stress with more positive emotions, adjust their emotional state in a timely manner, and correctly deal with the loss of resources (Scheier and Carver, 1993). As such, even though optimistic employees are exposed to abusive supervision variability, they can more effectively handle such stressful work experiences and the level of psychological detachment is less affected. In other words, optimism can moderate the relationship between abusive supervision variability and psychological detachment and the indirect effect of abusive supervision variability on work–family conflict through psychological detachment.

The current study makes several contributions to the literature. First, this study extends the literature on the impact of abusive supervision variability from the work domain to the non-work domain. Previous research has mainly focused on the work-related effects of abusive supervision variability but ignored its potential impacts on non-work outcomes. This study investigates how abusive supervision variability affects work–family conflict and could contribute to a more comprehensive understanding of the effects of abusive supervision variability. Second, this study enriches the understanding of the mechanism underlying abusive supervision variability. Extant studies have mainly relied on social identity theory and attachment theory to explore the mechanisms through which abusive supervision variability affects work-related outcomes. Differently, this study builds upon the conservation of resources theory to identify psychological detachment as a key mediator linking abusive supervision variability and work–family conflict, which provides a new theoretical angle for investigating the mechanism of abusive supervision variability. Third, this study enlarges the understanding of the boundary conditions of the effect of abusive supervision variability on outcomes. Previous studies have examined the moderating role of social exchange quality and perception of justice on the relationship between abusive supervision variability and work-related outcomes. This study explores the moderating role of employee optimism in the relationship between abusive supervision variability and psychological detachment, as well as the indirect relationship between abusive supervision variability and work–family conflict through psychological detachment. This not only could contribute to the theoretical understanding of the contextual factors of the effects of abusive supervision but also could provide novel insight on how to intervene in the effects of abusive supervision variability.

## Literature review and hypothesis development

### Abusive supervision variability and work–family conflict

Abusive supervision variability refers to leaders in the same team carrying out differential abuse toward different subordinates, such as deliberately mocking, belittling, and snubbing specific subordinates. Such differentiated and targeted abusive supervision makes individuals more aware of the influence of abusive supervision (Ogunfowora, 2013). Work–family conflict refers to the lack of time allocated to the family due to role conflict or overinvestment in work, resulting in an imbalance between the satisfaction from meeting work and family needs (Netemeyer et al., 1996; Lin et al., 2013) and creating conflict.

When individuals suffer from abusive supervision variability, they lose the support of their superiors for their work and need to spend more time and energy attempting to guess at the requirements of their leaders to meet their working standards and at the intention and reasons for leaders’ different abuse behaviors (Farh and Chen, 2014; Ogunfowora et al., 2019). From the perspective of the conservation of resources theory, abusive supervision variability is an important source of work stress (Tang et al., 2014), and individuals need to expend substantial resources to address such pressure. When abusive supervision variability persists, individuals are prone to depression and psychological exhaustion when they are under high pressure and resource depletion for a long time (Peng et al., 2019), causing them to be unable to happily participate in family activities (Bakker et al., 2008; Carlson et al., 2011; Lim and Lee, 2011). Even when individuals are faced with resource desperation, they attack others and engage in other irrational behaviors (Halbesleben et al., 2014). Thus, we believe that abusive supervision variability may affect individuals’ work and family lives, creating individual work–family conflict. Accordingly, we propose:

*H1*: Abusive supervision variability is positively related to individual work–family conflict.

### Abusive supervision variability and psychological detachment

Psychological detachment refers to the state in which an individual no longer carries out work-related affairs or thinks about the work during non-working hours and is physically and psychologically relaxed (Sonnentag and Fritz, 2007; Sonnentag et al., 2010). In general, after leaving the workplace, individuals disengage from the busy state of work through leisure activities and achieve psychological detachment by staying away from work pressure to supplement their resources lost during daily work (Fritz et al., 2010; Chen and Liu, 2019). Similarly, individuals affected by excessive or continuous work pressure have difficulty breaking away from their work state to a certain extent, and continuous work pressure causes a low level of psychological detachment and an inability to recover resources through leisure activities (Sonnentag et al., 2010). Work pressure has a serious negative impact on individuals’ degree of psychological detachment (Jiang et al., 2020).

As seen earlier, when individuals suffer from abusive supervision variability, they experience loss of work support, self-doubt, and shame, which creates great psychological pressure for them (Smith et al., 1998; Schaubroeck et al., 2016). Great psychological pressure leads to the dilemma in which individuals cannot completely disengage their body and mind from work, seriously affecting the high level of psychological detachment needed to recover their resources. Some scholars have found that excessive work pressure causes excessive loss of individual resources and emotional exhaustion, which have significant impacts on an individual’s degree of psychological detachment (Ma et al., 2014), suggesting the following hypothesis:

*H2*: Abusive supervision variability is negatively related to psychological detachment.

### Psychological detachment and work–family conflict

Individual psychological detachment is closely related to individual resource recovery. After work, individuals stop thinking about work, achieve psychological detachment by staying away from work pressure (Sonnentag and Bayer, 2005), and recover and supplement the resources they consume during work (Fritz et al., 2010). Individuals’ low psychological detachment prevents quick recovery and consumes resources because of the interference from continuous work pressure, causing fatigue and even emotional exhaustion. The individual’s work life strongly penetrates his or her family life (Edwards and Rothbard, 2000). When employees are in a stage of low psychological detachment, it is difficult for them to have sufficient cognitive and emotional resources to share with their family life and to accompany their family (Jiang et al., 2020), resulting in family dissatisfaction. Meanwhile, it is also difficult for individuals to control themselves and not express strong aggression toward their families due to a lack of sufficient cognitive and emotional resources, causing family conflict to arise (Wang et al., 2019). A high level of psychological detachment can help individuals maintain positive emotions and reduce the risk of work–family conflict to a certain extent (Gong et al., 2012). Thus, the theory and existing evidence lead us to propose the following hypothesis:

*H3*: Psychological detachment is negatively related to work–family conflict.

The conservation of resources theoretical logic suggests that psychological detachment is likely to mediate the relationship between abusive supervision variability and work–family conflict. First, when individuals suffer from abusive supervision variability from leaders in the workplace, they must spend a lot of cognitive and emotional resources to cope with it, and abusive supervision variability causes individuals greater psychological pressure (Ogunfowora, 2013), resulting in individuals unable to get out of work. In non-working hours, they are constantly disturbed by work and unable to engage in leisure activities in non-work fields (Tang et al., 2014), which leads to a low level of individual psychological detachment (Sonnentag et al., 2010; Jiang et al., 2020). Therefore, this study proposes the hypothesis that abusive supervision variability is negatively related to individual psychological detachment.

Second, when individuals are in a state of low-level psychological detachment, they are unable to completely get out of work and be disturbed by affairs in the field of work during non-working hours, which makes it difficult for them to have enough energy to accompany their families and affect the recovery of their own resources. Individual work and family field is inseparable and has a strong permeability. When individuals are in a low level of psychological detachment, they can easily transmit negative emotions from work to family life, causing dissatisfaction among family members and resulting in work–family conflict (Frenkel et al., 2011; Oosthuizen et al., 2011; Ma et al., 2014). Therefore, this study proposes the hypothesis that psychological detachment is negatively related to individual work–family conflict.

In conclusion, abusive supervision variability increases the risk of work–family conflict by reducing psychological detachment. Therefore, this study proposes that psychological detachment mediates abusive supervision variability and work–family conflict. The preceding discussion leads us to hypothesize that:

*H4*: Psychological detachment mediates the relationship between abusive supervision variability and work–family conflict.

### Moderating role of optimism

Our mediating model above states that abusive supervision variability leads to more stress and lower levels of psychological detachment. Such a low level of psychological detachment fosters the failure of the timely recovery of individual resources (Carlson et al., 2011), resulting in work–family conflicts. Following the same theoretical lens, we further investigate how optimism plays a moderating role in this process.

Based on the logic of the conservation of resources theory, individuals’ optimism may weaken the negative impact of abusive supervision variability on psychological detachment. First, individuals with strong optimism can quickly recover from negative emotions when facing high-level, destructive changes, generate more positive emotions, and adopt positive ways to address destructive stress as much as possible (Carver and Scheier, 2014). Therefore, when encountering abusive differences, they tend to resolve the difficulties caused by superiors in a more positive way, more effectively accomplish their work and reduce psychological resource consumption.

Second, individuals with strong optimism tend to have a stronger sense of purpose, persevere in high-pressure situations (Nes and Segerstrom, 2006), and easily achieve career success. They do not give up their planned career goals because of the immediate difficulties and unremittingly find a way to “break the situation” of existing setbacks (Peterson et al., 1988). Even if abusive supervision availability creates work pressure on individuals, those with high optimism still focus on their career goals, self-replenish resource losses, and adjust their working state of mind in a timely manner. Therefore, optimism can alleviate the impact of work pressure on psychological detachment.

Third, individuals with strong optimism easily obtain better interpersonal relationships and more support from their surroundings. Optimists are good at dealing with complex interpersonal problems (Smith et al., 2013) and are more likely to obtain better interpersonal relationships and support from others. Therefore, although individuals must face greater work pressure when experiencing abusive supervision variability, they can easily obtain support from colleagues, relatives, and friends, recover their own resources in a timely manner, and avoid affecting the level of psychological detachment due to excessive work pressure. We, therefore, predict that optimism moderates the relationship between abusive supervision variability and psychological detachment, as per the following hypothesis.

*H5a*: Optimism moderates the negative relationship between abusive supervision variability and psychological detachment. The relationship is stronger at higher levels of optimism.

**Figure 1:**
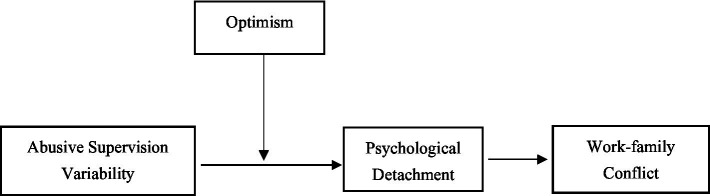
Overall research model.

Based on the earlier analysis, optimism plays a moderating role between abusive supervision variability and psychological detachment. Existing studies have proven a significant positive correlation between individual positive emotions and psychological detachment (Peng C. et al., 2014). Individuals more easily maintain positive emotions when they experience a high degree of psychological detachment. As a kind of positive emotion, optimism affects the mediating effect of psychological detachment on work–family conflict by influencing the degree of psychological detachment. When facing the work pressure caused by abusive supervision variability, individuals with strong optimism more easily attach a higher level of psychological detachment, alleviating the impact on their work–family conflict (Figure 1), suggesting the following hypothesis:

*H5b*: Optimism moderates the mediating effect of abusive supervision variability on work–family conflict through psychological detachment.

## Method

### Samples and procedures

Our sample consisted of employees working in different departments of nine companies covering real estate, construction, intelligent manufacturing, and finance in Guangdong Province, PRC. The surveys were conducted by an online system at two-time points to reduce the concern of common method variance (CMV; Podsakoff et al., 2012). At time 1 (T1), we invited 400 employees to participate in the survey. They were invited to report their demographic information, provide the last six digits of the mobile phone number and evaluate their perception of abusive supervision variability, psychological detachment, and optimism. Before answering the questionnaires, the participants were notified that they could do the survey on a voluntary basis and that their responses would be kept strictly confidential and for the only purpose of academic research.

We finally received 362 questionnaires (response rate: 90.5%). At time 2 (T2), 1 month after T1, participants who responded at T1 were asked to evaluate their work–family conflict and provide the last six digits of the mobile phone number. We obtained 352 questionnaires in this round data collection (response rate: 97.3%). The questionnaire was valid only after the last six digits of the mobile phone number were verified and consistent two times. The final sample consists of 260 responses (response rate: 65.0%). Of the respondents in the final sample, 51.4% were men, and 69.1% were between 26 and 35 years of age. A total of 93.8% had a college degree or above, and 87.95% held entry-level positions.

### Measurement

#### Abusive supervision variability

We measured abusive supervision variability using the Ogunfowora (2019) seven-item abusive supervision variability measure. Sample items were “My team leader ridicules some group members but not others” and “My team leader puts specific group members down in front of others.” Participants rated their leaders on a scale from 1 (strongly disagree) to 7 (strongly agree; α = 0.99).

#### Work–family conflict

Work–family conflict was measured at time 2 using five items adapted from a scale developed by Netemeyer et al. (1996). Sample items were “My family life is often disrupted by work demands” and “My work takes up so much of my time that I cannot properly perform family responsibilities.” Participants rated their work and family conflict on a scale from 1 (strongly disagree) to 7 (strongly agree; α = 0.97).

#### Psychological detachment

The psychological detachment was measured using four items developed by Sonnentag and Fritz (2007). Sample items were “I distanced myself from my work” and “I got a break from the demands of work.” Participants rated their psychological detachment on this scale from 1 (strongly disagree) to 7 (strongly agree; α = 0.98).

#### Optimism

We assessed optimism with the six-item optimism scale developed by Luthans (2007). Sample items were “When things can be good or bad, I usually think the outcome will be good” and “I always see the good in my work.” Participants rated their work and family conflict on a scale from 1 (strongly disagree) to 7 (strongly agree; α = 0.96).

#### Control variables

All analyses controlled for gender, age, education, position, and work tenure.

## Results

### Measurement model results

Since all the variables in the current study are self-rated, there might be a risk of CMV. To reduce CMV, we took two actions. First, an anonymous survey was adopted to reduce the suspicion and consistency tendency of the survey objects. Second, we collected data at two-time points with an interval of 1 month. We also tested whether our results were affected by CMV. The results show that the five-factor model with the common method deviation as the latent variable is not significantly better than the four-factor model (i.e., the theoretical model; ΔRMSEA = 0.0011 < 0.05, ΔSRMR = 0.0031 < 0.05, ΔCFI = 0.007 < 0.1, ΔTLI = 0.007 < 0.1). In addition, we followed Lindell and Whitney (2001) recommendations to test the CMV issue based on the marker variable approach. We identified education as a marker variable that is not relevant to the focal variables examined in this study (as shown by the low correlations of education with other focal variables in Table 1). We found that after controlling for CMV, the correlations of abusive supervision variability and psychological detachment with work–family conflict remained significant (*r* = 0.56 and *r* = 0.70, *p* < 0.01). The disattenuated partial correlations of the two variables with work–family conflict were slightly higher (*r* = 0.57 and *r* = 0.72).

**Table 1:** Means, standard deviations, and correlations between study variables (*N* = 260).

Variable	*M*	*SD*	1	2	3	4	5	6	7	8	9
1. Gender	1.49	0.50									
2. Age	2.07	0.70	−0.04								
3. Marital status	1.62	0.49	0.06	0.48**							
4. Education	2.78	0.79	0.00	−0.10	−0.10						
5. Working years	2.49	1.07	−0.01	0.70**	0.50**	−0.33**					
6. Position	1.16	0.47	−0.03	0.22**	0.11	0.11	0.25**				
7. Abusive supervision variability	3.39	2.01	−0.20**	0.19**	0.19**	−0.12	0.24**	−0.13*			
8. Psychological detachment	2.86	2.03	0.16**	−0.20**	−0.16*	0.02	−0.22**	−0.12	.-50**		
9. Optimism	4.78	1.47	0.07	−0.03	−0.04	0.15*	−0.15*	0.12	−0.16*	0.10	
10. Work–family conflict	3.60	2.10	−0.12	−0.14	−0.04	−0.10	−0.22**	−0.02	0.57**	−0.71**	−0.14*

*N* = 260, ***p < 0.001, **p < 0.01, *p < 0.05, the correlation coefficient is the Pearson coefficient (two-tailed test).

We also conducted a series of confirmatory factor analyses (CFAs) to test the distinctiveness between the key variables. We compared the hypothesized four-factor model to the alternative three-factor, two-factor, and one-factor models. The results (see Table 2) show that the hypothesized four-factor model was superior in fit to the other models and indicate that the four variables involved in this study were distinctive and represented four different constructs.

**Table 2:** Confirmatory factor analysis results.

Model	*χ* ^2^	df	*χ*^2^/*df*	RMSEA	CFI	TLI
Four-factor model: ASV;PD;O;WFC	398.04	203.00	1.96	0.06	0.98	0.98
Three-factor model: ASV + O;PD;WFC	2110.92	206.00	10.25	0.19	0.81	0.79
Three-factor model: ASV;O;PD + WFC	1556.91	206.00	7.56	0.16	0.87	0.85
Two-factor model: ASV + PD + O;WFC	3675.60	208.00	17.67	0.25	0.65	0.62
Two-factor model: ASV;PD + O + WFC	3278.07	208.00	15.76	0.24	0.69	0.66
One-factor model: ASV + PD + O + WFC	5717.66	209.00	27.36	0.32	0.45	0.39

ASV represents abusive supervision variability, PD represents psychological detachment, O represents optimism, WFC represents work–family conflict, and “+” represents two factors combined into one factor; *N* = 260.

### Hypothesis tests

Table 1 shows the mean, standard deviation, and correlation coefficient of each variable in the study. Abusive supervision variability was significantly positively correlated with work–family conflict (*r* = 0.57, *p* < 0.01) and significantly negatively correlated with psychological detachment (*r* = −0.50, *p* < 0.01). The psychological detachment was negatively correlated with work–family conflict (*r* = −0.71, *p* < 0.01).

Hypothesis 1 predicted that abusive supervision variability would be positively related to work–family conflict. As shown in Table 3, the effect of abusive supervision variability on work–family conflict was significant and positive (*β* = 0.30, *t* = 4.67, *p* < 0.001). Thus, hypothesis 1 was supported.

**Table 3:** Model testing result.

Variables	Psychological detachment		Work–family conflict			
	*B*	*SE*	*t*	*B*	*SE*	*t*
Intercept	1.33	0.73	1.83	4.45***	0.56	7.95
**Control variable**						
Gender	0.18	0.23	0.75	0.20	0.18	1.15
Age	−0.13	0.23	−0.55	−0.06	0.17	−0.34
Marital status	−0.01	0.26	−0.04	−0.71***	0.22	−3.21
Education	−0.11	0.15	−0.71	−0.07	0.12	−0.58
Working years	−0.05	0.17	−0.30	0.21	0.13	1.68
Position	−0.73***	0.22	−3.25	−0.22	0.27	−0.80
**Independent variable**						
Abusive supervision variability	−0.48***	0.06	−8.15	0.30***	0.06	4.67
**Mediating variable**						
Psychological detachment				−0.58***	0.06	−10.30
**Moderator**						
Optimism	0.02	0.08	0.20	0.01	0.06	0.19
**Interaction**						
Abusive supervision variability × Optimism	0.09*	0.04	2.39			
*R* ^2^	0.31***		0.60***			

*N* = 260, ***p < 0.001, **p < 0.01, *p < 0.05; the correlation coefficient is the Pearson coefficient (two-tailed test).

Hypothesis 2 predicted that abusive supervision variability would be positively related to psychological detachment. The results in Table 3 show that the effect of abusive supervision variability on psychological detachment was significant and negative (*β* = −0.48, *t* = −8.15, *p* < 0.001). Thus, hypothesis 2 was supported.

Hypothesis 3 predicted that psychological detachment would be negatively related to work–family conflict. The results show that psychological detachment was significantly and negatively related to work–family conflict (*β* = −0.58, *t* = −10.30, *p* < 0.001). Thus, hypothesis 3 was supported.

Hypothesis 4 predicted the mediating effect of psychological detachment between abusive supervision variability and work–family conflict. As shown in Table 4, the indirect effect value was 0.28, the lower limit of the 95% unbiased confidence interval was 0.20, and the upper limit was 0.38, excluding 0; therefore, psychological detachment mediated the relationship between abusive supervision variability and work–family conflict effects.

**Table 4:** Summary of mediation effect and moderated mediation effect results.

	Effect Value	SE	Indirect effect
			95% Unbiased signal interval
Mediation effect	0.28	0.05	[0.20, 0.38]
Moderated mediation effects			
High Optimistic	0.21	0.05	[0.12, 0.31]
Low Optimistic	0.35	0.06	[0.24, 0.48]
Difference	−0.15	0.07	[−0.28, −0.02]

*N* = 260, bootstrapping sample number is 5000 ***p < 0.001, **p < 0.01, *p < 0.05. The correlation coefficient is the Pearson coefficient (two-tailed test).

Hypothesis 5a predicted that optimism would moderate the negative relationship between abusive supervision variability and psychological detachment. The relationship was stronger at higher levels of optimism.

As shown in Table 3, the interaction of abusive supervision variability and optimism positively predicted psychological detachment (*β* = 0.09, *t* = 2.39, *p* < 0.05). This result indicated that optimism plays a moderating role between abusive supervision variability and psychological detachment. Thus, hypothesis 5a was supported.

**Figure 2:**
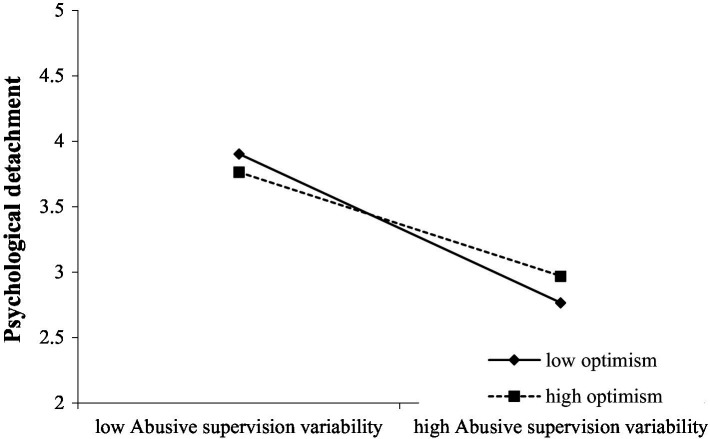
Interaction of abusive supervision variability and optimism in predicting psychological detachment.

To further analyze the moderating role of optimism, this study plots the moderating effect of optimism, as shown in Figure 2. Figure 2 shows that when optimism is higher, the positive relationship between abusive supervision variability and psychological detachment is weaker, and vice versa. The research hypothesis 5a was further verified.

Hypothesis 5b predicted that optimism would moderate the mediating effect of abusive supervision variability on work–family conflict through psychological detachment. As shown in Table 4, the difference value between the higher optimism condition and lower optimism condition was −0.15, and the 95% unbiased confidence interval was [−0.28, −0.02], excluding 0, indicating that there is a moderated mediation effect. Therefore, research hypothesis 5b was supported.

## Discussion

The goal of our research was to examine whether and how abusive supervision variability influences employees’ work–family conflicts. The results of our study show that abusive supervision variability is positively related to work–family conflict. Abusive supervision variability was negatively related to psychological detachment. Moreover, the psychological detachment was negatively related to work–family conflict. Psychological detachment played a mediating role in the relationship between abusive supervision variability and work–family conflict. Optimism moderates the indirect effect of abusive supervision variability on work–family conflict through psychological detachment such that the indirect effects are weaker when optimism is higher rather than lower.

### Theoretical implications

First, this study expands the research on abusive supervision variability from the work domain to the non-work domain by exploring its relationship with employees’ work–family conflicts. Work and family are an indispensable part of the life of every employee and even all human beings. The existing literature shows employees’ depression caused by work pressure relieves their depression by relating it to their families or taking revenge on them (Hoobler and Brass, 2006). However, this usually does not help employees effectively relieve their bad emotions. On the contrary, employees may feel guilt toward their families or further strengthen these bad emotions due to unpleasant interactions with their families (Van Woerkom and Meyers, 2015). With these negative emotions, it is difficult for employees to devote themselves to their work efficiently, which affects their work performance and thus creates a vicious cycle (Liu et al., 2015). Therefore, if we focus on only the impact of abusive supervision variability on employees’ performance in the workplace and ignore its possible impact on their family life, it will lead us to underestimate the negative impact of abusive supervision variability and create a lack of theoretical knowledge. Based on the conservation of resources theory, this study explores the cross-domain effects of abusive supervision variability as a stressor on individual employees in the family domain and verifies this hypothesis through empirical analysis, which enriches the understanding of the implication of abusive supervision variability at the theoretical level.

Second, this study also deepens the understanding of the mechanism of abusive supervision variability by using the conservation of resources theory to identify that psychological detachment is an important mediator. Existing studies have explored the mediating mechanism of abusive supervision variability affecting employees’ performance in the workplace from the theoretical perspectives of social identity theory, attachment theory, and emotional cognitive evaluation theory (Schaubroeck et al., 2016; Ogunfowora et al., 2019; Peng et al., 2019). However, these theoretical perspectives focus on analyzing the impact of stress experienced by employees in the workplace on their work performance, which cannot determine whether or how abusive supervision variability spill over into employees’ family lives. Based on the conservation of resources theory, this study argues that abusive supervision variability leads to individuals’ inability to obtain high levels of psychological detachment, while low levels of psychological detachment do not allow individuals to achieve self-resource recovery or develop work–family conflict. Thus, psychological detachment is the core mechanism through which abusive supervision variability is related to work–family conflict. This not only broadens our understanding of the mechanisms underlying the effects of abusive supervision variability but also provides a new analytical framework for analyzing the implications of abusive supervision variability. Moreover, this analytical framework can also be used to analyze the effects of other stress-related workplace experiences on individuals’ family lives. For example, when employees become the target of negative gossip and perceive greater psychological stress in the workplace, it can lead to an inability to fully detach from work, affecting their status in family life and creating adverse interactions with their families, which eventually leads to work–family conflict.

Third, the study extends the literature on abusive supervision variability by exploring the moderating role of optimism in the relationship between abusive supervision variability and work–family conflict. Previous studies have used the attitude of third-party employees as a moderating variable and have pointed out that abusive differences stimulate employees to produce moral responses and then jointly resist the abusive behavior of leaders (Mitchell et al., 2015). Some scholars use inter-team and intra-team competitive atmospheres as moderating variables to study the moderation of the relationship between abusive supervision variability and team performance (Wang, 2017). However, none of these moderating variables started from the individuals themselves; thus, it is important to explore the role played by individual traits in the effects of abusive supervision variability. This study explores the moderating role of individual optimism in the relationship between abusive supervision variability and psychological detachment at the individual level and applies optimism, a common research topic in the field of psychology, to management phenomena, enriching the research content on the contingency factors of abusive supervision variability to a certain extent.

Fourth, this study enriches the research on the antecedents of work–family conflict. Previous studies on work–family conflict have shown that role conflict and insufficient role-playing are important factors that stimulate work–family conflict. However, this study shows that the substantial pressure placed on employees by workplace unfairness, such as abusive supervision variability, makes it difficult for employees to achieve psychological detachment during non-working hours, thus triggering work–family conflict. This finding also provides a new perspective for the study of the antecedents of work–family conflict.

### Practical implications

This study provides meaningful insights into management practices.

First, management practitioners should pay attention to the negative implication of abusive supervision variability on employees’ family lives. Our results show that abusive supervision variability stimulates work–family conflict. Employees may experience guilty over their families or further strengthen these negative emotions due to unpleasant family interactions (Van Woerkom and Meyers, 2015). When experiencing these negative emotions, it is difficult for employees to efficiently devote themselves to work, which brings poor work performance, thus creating a vicious circle (Liu et al., 2012). In this regard, supervisors should reduce or even eliminate improper management behavior, such as abusive supervision variability, to improve employees’ family life quality and work efficiency.

Second, managers should attach importance to the mediation of psychological detachment. Combined with what has been demonstrated in this study, abusive supervision variability can directly make it difficult for employees to get off-work and get adequate rest, creating work–family conflict. It is necessary to adopt methods, such as increasing staff leisure activities and paying attention to employees’ mental health and emotional management to help them distinguish the boundaries between work and family life. This could help employees improve their psychological detachment and better achieve a balance between work and family (Wu et al., 2020), thus reducing the negative implication of abusive supervision variability on work–family conflict.

Third, based on our findings regarding the moderating role of optimism, organizations should select employees with optimistic characteristics and provide relevant support to improve their optimism. High optimism can alleviate the negative implication of abusive supervision variability on employees. Optimists find ways to solve problems when under stress (Carver et al., 2010). Therefore, managers should take appropriate measures to help employees improve their optimism, such as providing professional consultations and creating a positive organizational atmosphere.

### Limitations and future research directions

Some limitations in our study should be noted. First, subordinates provided perceived ratings on independent variables, mediators and moderators, and dependent variables. Even though we measured these variables at two-time points, our results may be affected by CMV. Future research should collect data from different sources. We cannot unequivocally claim the causality in the relationships among abusive supervision variability, phycological detachment, and work–family conflicts. Longitudinal or experimental research designs can be adopted in future research to establish causality and identify the change effects of the constructs over time.

Second, all our data were collected in South China. We are not sure whether the results obtained in this study can be generalized to other contexts. We suggest future studies to test the model with a large-scale sample of employees from multiple regions and countries, which could improve the accuracy of the analysis.

Third, this study proposes and tests the moderating role of only optimism. Future studies should further examine other potential moderators. Existing studies have noted that perceived organizational support can weaken the impact of stress on employees’ negative cognitions and emotions (Rhoades et al., 2001; Han and Liu, 2009; Liu et al., 2020). Based on this logic, perceived organizational support may also weaken the negative effects of abusive supervision variability on work–family conflict. Therefore, we suggest that future research should explore other potential boundary conditions that could affect the relationship between abusive supervision variability and employees’ work–family conflicts at the organizational level.

## Conclusion

Our findings indicate that employees who suffer from abusive supervision variability cannot obtain a high level of psychological detachment, which, in turn, causes work–family conflict. Optimism can enable employees to experience more positive emotions, thus supplementing resources in a timely manner and alleviating the negative impact of abusive supervision variability on psychological detachment as well as the indirect effect of abusive supervision variability on work–family conflict through psychological detachment. These findings extend the literature on the effect of abusive supervision variability from the work domain to the non-work domain and provide valuable insights for practitioners on how to intervene in the negative effects of abusive supervision variability.

## Data availability statement

The original contributions presented in the study are included in the article/supplementary material, further inquiries can be directed to the corresponding author.

## Ethics statement

Ethical review and approval was not required for the study on human participants in accordance with the local legislation and institutional requirements. Written informed consent from the (patients/participants OR patients/participants legal guardian/next of kin) was not required to participate in this study in accordance with the national legislation and the institutional requirements.

## Author contributions

SW: conceptualization and writing original draft. XL: data curation, performed analysis, and writing original draft. JW: conceptualization, supervision, and writing review and editing. All authors contributed to the article and approved the submitted version.

## Funding

This study was funded by Guangzhou Education Bureau Innovation and Entrepreneurship Education Project (2019PT102) and Guangzhou key Research Base of Humanities and Social Sciences “Guangzhou Research Base for Cooperative training of High-tech talents” (Base No. 2 Co-construction of key Research Base).

## Conflict of interest

The authors declare that the research was conducted in the absence of any commercial or financial relationships that could be construed as a potential conflict of interest.

## Publisher’s note

All claims expressed in this article are solely those of the authors and do not necessarily represent those of their affiliated organizations, or those of the publisher, the editors and the reviewers. Any product that may be evaluated in this article, or claim that may be made by its manufacturer, is not guaranteed or endorsed by the publisher.

## References

[ref1] Bakker A. B. Demerouti E. Dollard M. F. (2008). How job demands affect partners' experience of exhaustion: integrating work-family conflict and crossover theory. J. Appl. Psychol. 93, 901–911. doi: 10.1037/0021-9010.93.4.901, PMID: 18642992

[ref2] Burton J. P. Taylor S. G. Barber L. K. (2014). Understanding internal, external, and relational attributions for abusive supervision. J. Organ. Behav. 35, 871–891. doi: 10.1002/job.1939

[ref3] Carlson D. S. Ferguson M. Perrewé P. L. Whitten D. (2011). The fallout from abusive supervision: an examination of subordinates and their partners. Pers. Psychol. 64, 937–961. doi: 10.1111/j.1744-6570.2011.01232.x

[ref01] Carver C. S. Scheier M. F. Segerstrom S. C. (2010). Optimism. Clinical Psychology Review 30, 879–889. doi: 10.1016/j.cpr.2010.01.006 20170998 PMC4161121

[ref4] Carver C. S. Scheier M. F. (2014). Dispositional psychological detachment. Trends Cogn. Sci. 18, 293–299. doi: 10.1016/j.tics.2014.02.003, PMID: 24630971 PMC4061570

[ref5] Chen S. C. Liu N. T. (2019). When and how vicarious abusive supervision leads to bystanders’ supervisor-directed deviance: a moderated–mediation model. Pers. Rev. 48, 1734–1755. doi: 10.1108/PR-09-2018-0368

[ref6] Choi W. Kim S. L. Yun S. (2019). A social exchange perspective of abusive supervision and knowledge sharing: investigating the moderating effects of psychological contract fulfillment and self-enhancement motive. J. Bus. Psychol. 34, 305–319. doi: 10.1007/s10869-018-9542-0

[ref7] Edwards J. R. Rothbard N. P. (2000). Mechanisms linking work and family: clarifying the relationship between work and family constructs. Acad. Manag. Rev. 25, 178–199. doi: 10.2307/259269

[ref8] Farh C. I. Chen Z. (2014). Beyond the individual victim: multilevel consequences of abusive supervision in teams. J. Appl. Psychol. 99, 1074–1095. doi: 10.1037/a0037636, PMID: 25111251

[ref9] Foulk T. A. Lanaj K. Tu M. H. Erez A. Archambeau L. (2018). Heavy is the head that wears the crown: an actor-centric approach to daily psychological power, abusive leader behavior, and perceived incivility. Acad. Manag. J. 61, 661–684. doi: 10.5465/amj.2015.1061

[ref53] Frenkel S. J. Li M. Restubog S. L. (2011). Management, organizational justice and psychological detachment among Chinese migrant workers: evidence from two manufacturing firms. Br. J. Ind. Relat. 50, 121–147. doi: 10.1111/j.1467-8543.2011.00858.x

[ref10] Fritz C. Sonnentag S. Spector P. E. McInroe J. A. (2010). The weekend matters: relationships between stress recovery and affective experiences. J. Organ. Behav. 31, 1137–1162. doi: 10.1002/job.672

[ref11] Gong H. Wang Y. L. Lu J. H. (2012). Free from work? The role of psychological relief in the work/family interface. CUHK Manage. Res. 7:20.

[ref02] Halbesleben J. R. Neveu J. P. Paustian-Underdahl S. C. Westman M. (2014). Getting to the “COR” undertanding the role of resources in conservation of resources theory. Journal of Management 40, 1334–1364. doi: 10.1177/01492063145271

[ref13] Han Y. Liu J. Z. (2009). Individual organization fit, organizational support and turnover intention -- the mediating role of job satisfaction. Econ. Manag. 2:8.

[ref14] Hannah S. T. Schaubroeck J. M. Peng A. C. Lord R. G. Trevino L. K. Kozlowski S. W.. (2013). Joint influences of individual and work unit abusive supervision on ethical intentions and behaviors: a moderated mediation model. J. Appl. Psychol. 98, 579–592. doi: 10.1037/a0032809, PMID: 23647209

[ref15] Harris K. J. Harvey P. Kacmar K. M. (2011). Abusive supervisory reactions to coworker relationship conflict. Leadersh. Q. 22, 1010–1023. doi: 10.1016/j.leaqua.2011.07.020

[ref16] Harvey P. Stoner J. Hochwarter W. Kacmar C. (2007). Coping with abusive supervision: the neutralizing effects of ingratiation and positive affect on negative employee outcomes. Leadersh. Q. 18, 264–280. doi: 10.1016/j.leaqua.2007.03.008

[ref17] Hobfoll S. E. Halbesleben J. Neveu J. P. Westman M. (2018). Conservation of resources in the organizational context: the reality of resources and their consequences. Annu. Rev. Organ. Psych. Organ. Behav. 5, 103–128. doi: 10.1146/annurev-orgpsych-032117-104640

[ref18] Hoobler J. M. Brass D. J. (2006). Abusive supervision and family undermining as displaced aggression. J. Appl. Psychol. 91:1125. doi: 10.1037/0021-9010.91.5.1125, PMID: 16953773

[ref19] Hu J. Zheng X. Tepper B. J. Li N. Liu X. Yu J. (2022). The dark side of leader–member exchange: Observers' reactions when leaders target their teammates for abuse. Hum. Resour. Manag. 61, 199–213. doi: 10.1002/hrm.22088

[ref20] Huang J. Yan L. Luo S. Q. Wu Y. Q. Luo R. J. (2022). Research and evaluation on abusive supervision from third-party perspective-big data visualization and analysis based on cite space. Soft Sci. 5, 1–13.

[ref21] Jiang W. Gu Q. (2016). How abusive supervision and abusive supervisory climate influence salesperson creativity and sales team effectiveness in China. Manag. Decis. 54, 455–475. doi: 10.1108/MD-07-2015-0302

[ref22] Jiang W. Gu Q. Tang T. L. P. (2019). Do victims of supervisor bullying suffer from poor creativity? Social cognitive and social comparison perspectives. J. Bus. Ethics 157, 865–884. doi: 10.1007/s10551-017-3660-x

[ref23] Jiang J. W. Li M. L. Zhou Q. N. (2020). The impact of job insecurity on work family conflict -- an analysis of the mediating role of regulation. Hum. Resour. Manag. Rev. 1:13.

[ref24] Liao Z. Lee H. W. Johnson R. E. Song Z. Liu Y. (2021). Seeing from a short-term perspective: when and why daily abusive supervisor behavior yields functional and dysfunctional consequences. J. Appl. Psychol. 106, 377–398. doi: 10.1037/apl0000508, PMID: 32352822

[ref25] Lim S. Lee A. (2011). Work and nonwork outcomes of workplace incivility: does family support help? J. Occup. Health Psychol. 16:95. doi: 10.1037/a0021726, PMID: 21280947

[ref03] Lin Z. Ju L. Chen L. (2013). Research on Work-Family conflict and Chinese issues: Perspective. Content and Design Management World 9, 154–171.

[ref04] Lindell M. K. Whitney D. J. (2001). Accounting for common method variance in cross-sectional research designs. Journal of Applied Psychology 86:114. doi: 10.1037/0021-9010.86.1.114 11302223

[ref26] Liu D. Liao H. Loi R. (2012). The dark side of leadership: a three-level investigation of the cascading effect of abusive supervision on employee creativity. Acad. Manag. J. 55, 1187–1212. doi: 10.5465/amj.2010.0400

[ref27] Liu L. Mei Q. Wu J. N. (2020). Employee well-being, job stress and innovation behavior: the regulatory role of perceived organizational support. Sci. Technol. Prog. Countermeasures 37:7.

[ref28] Liu Y. Wang M. Chang C. H. Shi J. Zhou L. Shao R. (2015). Work–family conflict, emotional exhaustion, and displaced aggression toward others: the moderating roles of workplace interpersonal conflict and perceived managerial family support. J. Appl. Psychol. 100, 793–808. doi: 10.1037/a0038387, PMID: 25528246

[ref05] Luthans F. Youssef C. M. Avolio B. J. (2007). Psychological capital: Developing the human competitive edge (Vol. *198*). Oxford: Oxford university press.

[ref29] Ma H. Xie J. Tang H. (2014). The relationship between organizational segmentation supply and work emotional exhaustion: the mediating role of work psychological detachment and work → non work conflict. Psychol. Behav. Res. 12, 527–532.

[ref06] Mawritz M. B. Mayer D. M. Hoobler J. M. Wayne S. J. Marinova S. V. (2012). A trickle-down modle of abusive supervision. Personnel Psychology 65, 325–357. doi: 10.1111/j.1744-6570.2012.01246.x

[ref30] Meyer J. P. Allen N. J. Smith C. A. (1993). Commitment to organizations and occupations: extension and test of a three-component conceptualization. J. Appl. Psychol. 78, 538–551. doi: 10.1037/0021-9010.78.4.538

[ref31] Mitchell M. S. Vogel R. M. Folger R. (2015). Third parties’ reactions to the abusive supervision of coworkers. J. Appl. Psychol. 100:1040. doi: 10.1037/apl0000002, PMID: 25243999

[ref32] Nes L. S. Segerstrom S. C. (2006). Dispositional psychological detachment and coping: a meta-analytic review. Personal. Soc. Psychol. Rev. 10, 235–251. doi: 10.1207/s15327957pspr1003_3, PMID: 16859439

[ref33] Netemeyer R. G. Boles J. S. McMurrian R. (1996). Development and validation of work–family conflict and family–work conflict scales. J. Appl. Psychol. 81, 400–410. doi: 10.1037/0021-9010.81.4.400

[ref34] Ogunfowora B. (2013). When the abuse is unevenly distributed: the effects of abusive supervision variability on work attitudes and behaviors. J. Organ. Behav. 34, 1105–1123. doi: 10.1002/job.1841

[ref35] Ogunfowora B. Weinhardt J. M. Hwang C. C. (2019). Abusive supervision differentiation and employee outcomes: the roles of envy, resentment, and insecure group attachment. J. Manag. 47, 623–653. doi: 10.1177/0149206319862024

[ref36] Oosthuizen J. Mostert K. Koekemoer F. E. (2011). Job characteristics, work-nonwork interference and the role of recovery strategies amongst employees in a tertiary institution. SA J. Hum. Resour. Manag. 9, 1–15. doi: 10.4102/sajhrm.v9i1.356

[ref37] Peng A. C. Schaubroeck M. Chong S. J. Li Y. (2019). Discrete emotions linking abusive supervision to employee intention and behavior. Pers. Psychol. 72, 393–419. doi: 10.1111/peps.12310

[ref38] Peng A. C. Schaubroeck J. M. Li Y. (2014). Social exchange implications of own and coworkers' experiences of supervisory abuse. Acad. Manag. J. 57, 1385–1405. doi: 10.5465/amj.2012.0080

[ref39] Peng C. Xie W. Li F. Ren L. (2014). Research progress of psychological detachment. Mod. Bus. 11:2.

[ref40] Peterson C. Seligman M. E. Vaillant G. E. (1988). Pessimistic explanatory style is a risk factor for physical illness: a thirty-five-year longitudinal study. J. Pers. Soc. Psychol. 55, 23–27. doi: 10.1037/0022-3514.55.1.23, PMID: 3418489

[ref41] Podsakoff P. M. MacKenzie S. B. Podsakoff N. P. (2012). Sources of method bias in social science research and recommendations on how to control it. Annu. Rev. Psychol. 63, 539–569. doi: 10.1146/annurev-psych-120710-100452 21838546

[ref42] Rhoades L. Eisenberger R. Armeli S. (2001). Affective commitment to the organization: the contribution of perceived organizational support. J. Appl. Psychol. 86, 825–836. doi: 10.1037/0021-9010.86.5.825, PMID: 11596800

[ref43] Rousseau V. Aubé C. (2018). When leaders stifle innovation in work teams: the role of abusive supervision. J. Bus. Ethics 151, 651–664. doi: 10.1007/s10551-016-3258-8

[ref44] Schaubroeck J. M. Peng A. C. Hannah S. T. (2016). The role of peer respect in linking abusive supervision to follower outcomes: dual moderation of group potency. J. Appl. Psychol. 101, 267–278. doi: 10.1037/apl0000050, PMID: 26348478

[ref46] Scheier M. F. Carver C. S. (1993). On the power of positive thinking: the benefits of being optimistic. Curr. Dir. Psychol. Sci. 2, 26–30. doi: 10.1111/1467-8721.ep10770572

[ref47] Shen C. Yang J. Hu S. (2020). Combined effect of abusive supervision and abusive supervision climate on employee creativity: a moderated mediation model. Front. Psychol. 11, 1–10. doi: 10.3389/fpsyg.2020.01175, PMID: 32754075 PMC7367141

[ref48] Smith T. W. Ruiz J. M. Cundiff J. M. Baron K. G. Nealey-Moore J. B. (2013). Psychological detachment and pessimism in social context: an interpersonal perspective on resilience and risk. J. Res. Pers. 47, 553–562. doi: 10.1016/j.jrp.2013.04.006, PMID: 27840458 PMC5102513

[ref49] Smith H. J. Tyler T. R. Huo Y. J. Ortiz D. J. Lind E. A. (1998). The self-relevant implications of the group-value model: group membership, self-worth, and treatment quality. J. Exp. Soc. Psychol. 34, 470–493. doi: 10.1006/jesp.1998.1360

[ref50] Song Y. Liu Y. Wang M. Lanaj K. Johnson R. E. Shi J. (2018). A social mindfulness approach to understanding experienced customer mistreatment: a within-person field experiment. Acad. Manag. J. 61, 994–1020. doi: 10.5465/amj.2016.0448

[ref51] Sonnentag S. Bayer U. V. (2005). Switching off mentally: predictors and consequences of psychological detachment from work during off-job time. J. Occup. Health Psychol. 10:393. doi: 10.1037/1076-8998.10.4.393, PMID: 16248688

[ref07] Sonnentag S. Fritz C. (2007). The Recovery Experience Questionnaire: development and validation of a measure for assessing recuperation and unwinding from work. J. Occup. Health Psychol. 12:204. doi: 10.1037/1076-892.3.204 17638488

[ref52] Sonnentag S. Binnewies C. Mojza E. J. (2010). Staying well and engaged when demands are high: the role of psychological detachment. J. Appl. Psychol. 95, 965–976. doi: 10.1037/a0020032, PMID: 20718528

[ref54] Tang G. Hu D. Wu L. Chen Y. (2014). Study on the influence of abusive supervision on employees' interpersonal deviant behavior and its mechanism. J. Manag. 11, 1782–1789.

[ref55] Tepper B. J. (2000). Consequences of abusive supervision. Acad. Manag. J. 43, 178–190. doi: 10.2307/1556375

[ref08] Tepper B. J. Henle C. A. Lambert L. S. Giacalone R. A. Duffy M. K. (2008). Abusive supervision and subordinates’ organization deviance. Journal of Applied Psychology 93:721. doi: 10.1037/0021-9010.93.4.721 18642979

[ref56] Tepper B. J. Simon L. Park H. M. (2017). Abusive supervision. Annu. Rev. Organ. Psych. Organ. Behav. 4, 123–152. doi: 10.1146/annurev-orgpsych-041015-062539

[ref57] Van Woerkom M. Meyers M. C. (2015). My strengths count! Effects of a strengths-based psychological climate on positive affect and job performance. Hum. Resour. Manag. 54, 81–103. doi: 10.1002/hrm.21623

[ref58] Wang Y. (2017). Research on the Impact of Abusive Supervision Varialbility on Team Performance in Team Competitive Atmosphere. Harbin, China: Harbin Institute of Technology.

[ref59] Wang T. Long L. Zhang J. Zhang Y. (2019). A study on the effect of family like exchange on work family conflict. Manag. Rev. 2:10.

[ref60] Wu H. Qiu S. Dooley L. M. Ma C. (2020). The relationship between challenge and hindrance stressors and emotional exhaustion: the moderating role of perceived servant leadership. Int. J. Environ. Res. Public Health 17:282. doi: 10.3390/ijerph17010282, PMID: 31906094 PMC6981731

[ref61] Zheng X. Liu X. (2017). The buffering effect of mindfulness on abusive supervision and creative performance: a social cognitive framework. Front. Psychol. 8, 1588–1600. doi: 10.3389/fpsyg.2017.01588, PMID: 28955285 PMC5600939

